# Screening of filamentous fungi for antimicrobial silver nanoparticles synthesis

**DOI:** 10.1186/s13568-017-0332-2

**Published:** 2017-02-01

**Authors:** Cristiane Angélica Ottoni, Marta Filipa Simões, Sara Fernandes, Jonas Gomes dos Santos, Elda Sabino da Silva, Rodrigo Fernando Brambilla de Souza, Alfredo Eduardo Maiorano

**Affiliations:** 1 Biosciences Institute, São Paulo State University-UNESP, Praça Infante Dom Henrique s/no, São Vicente, Parque Bitaru 11330-900 Brazil; 2 Biology Department, Edge Hill University, St Helens Road, Lancashire, Ormskirk, L39 4QP UK; 3 Richmond Pharmacology Ltd, St George’s University London, London, SW17 0RE England, UK; 4 Laboratório de Biotecnologia Industrial, Instituto de Pesquisa Tecnológica do Estado de São Paulo, São Paulo, Brazil; 5 Departamento de Química, Universidade Federal do Amazonas, Av. General Rodrigo Octávio, Coroado I, 6200, Manaus, AM 69080-900 Brazil

**Keywords:** Silver nanoparticles, Filamentous fungi, Mycogenic synthesis, Nitrate-reductase, Antimicrobial

## Abstract

The present work had the goal of screening a batch of 20 fungal strains, isolated from sugar cane plantation soil, in order to identify those capable of biosynthesis of silver nanoparticles. These nanoparticles are known to have a large and effective application in clinical microbiology. Four strains were found to be capable of biosynthesis of silver nanoparticles. The biosynthesised nanoparticles were characterised by UV–vis spectroscopy, scanning electron microscopy, EDX, and XRD. They were found to have an average size of 30–100 nm, a regular round shape, and potential antimicrobial activity against *Escherichia coli*, *Staphylococcus aureus,* and *Pseudomonas aeruginosa*. The antimicrobial activity was found to be directly related to the nanoparticles concentration. Mycogenic synthesis of nanoparticles is a green biogenic process preferable to other alternatives. Because fungi are great producers of extracellular enzymes this process makes scaling-up an easier task with high importance for clinical microbiology on the fight against microbial resistance, as well as for other industrial applications.

## Introduction

The rise of bacterial and fungal resistance against antimicrobials has promoted research of bactericidal nanomaterials, especially in the well-known area of silver ions and silver-based compounds, including silver nanoparticles (Jiravova et al. 2016; Monteiro et al. 2009). The latter have emerged as interesting antimicrobial agents due to their high surface-area-to-volume ratio and unique chemical and physical properties. They were previously described as “the largest and fastest growing category of nanotechnology-based medicines” (Chen et al. 2016), and provide a wide range of possible applications in areas as diverse as biomedical (prosthetics bone, surgical instruments), fashion (clothes and footwear production), beauty industry (conditioners, toothpaste), and clinical (for the treatment of wounds and infections) (Paschoalino et al. 2010; Durán et al. 2016). Their high demand makes it essential to develop environmentally benign procedures to synthesize silver nanoparticles for industrial and clinical purposes. A promising, reliable and eco-friendly approach is the use of natural sources and biological systems (Thakkar et al. 2010). A vast array of biological resources is available for this synthesis process, including: plants and plant products (Mittal et al. 2013), algae (Patel et al. 2015), fungi (Xue et al. 2016), yeast (Ortega et al. 2015) and bacteria (Pantidos and Horsfall 2014).

Among all biological resources, fungi present higher tolerance and metal bioaccumulation abilities, which are advantageous characteristics for the production of nanoparticles (Mandal et al. 2006). Another benefit of using fungi in nanoparticle synthesis is the ease in the scale-up which makes the entire process more cost-effective (Rahimi et al. 2016). Given that fungi are extremely efficient secretors of extracellular enzymes, it is thus possible to easily obtain large-scale production of nanoparticles (van den Hondel et al. 1992; Rahimi et al. 2016).

Also, exploring less studied environments can present new and different data on microbial diversity. Sugar cane plantation soil has not been thoroughly explored, but it has been reported that *Aspergillus* and *Rhizopus* are the dominant fungal genera present (Abdel-Rahim et al. 1983), and both have been described as being capable of AgNP synthesis (Banu et al. 2011; Zomorodian et al. 2016).

The underlying mechanisms of nanoparticles biosynthesis is yet to be fully elucidated. Although several factors acting together may determine the biological synthesis reaction, the identification of the most active biomolecules as reducing and stabilizing agents is essential in choosing the best technical parameters to be employed in the biosynthesis (Duschak 2016).

Biologically synthesized silver nanoparticles, are nontoxic for humans (in low concentrations), and safe inorganic antibacterial agents that have been shown to exhibit a strong toxicity to a wide range of microorganisms since ancient times (Shanthi et al. 2016; Roy et al. 2013; Annamalai and Nallamuthu 2016). It has been considered that AgNP mode of action depends on monovalent ionic silver (Ag^+^), which is released inside the microbial cells and inhibits microbial growth through suppression of respiratory enzymes and electron transport components (Li et al. 2006; Annamalai and Nallamuthu 2016; Chen et al. 2016). It has also been described that the AgNP affect the cellular membranes (Chen et al. 2016; Durán et al. 2016).

The present study aimed to: (1) identify different filamentous fungal strains capable of synthesizing silver nanoparticles (AgNP), (2) characterise the synthesised AgNP, and (3) analyse the antimicrobial activity of the produced nanoparticles against Gram-negative and -positive bacteria.

## Materials and methods

### Chemical compounds

Silver nitrate (PubChem CID: 24,470); sulphanilamide (PubChem CID: 5333); *N*-(1-naphthyl) ethylene diamine dihydrochloride (PubChem CID: 15,106); potassium nitrate (PubChem CID: 24,434); propanol (PubChem CID: 3776).

### Microorganisms

A batch of 20 different filamentous fungal strains (identified as: IPT825, IPT827, IPT829, IPT849, IPT853, IPT859, IPT868, IPT856, IPT1005, IPT1008, IPT1009, IPT1010, IPT1011, IPT1012, IPT1013, IPT1014, IPT1015, IPT1016, IPT1017, and IPT1018), previously isolated from sugar cane plantation soil, was supplied by Instituto de Pesquisa Tecnológica do Estado de São Paulo (IPT, São Paulo, Brazil). All strains were maintained on malt extract agar (MEA; 20 g/L malt extract, 20 g/L glucose, 1 g/L peptone and 15 g/L agar) as stock cultures at 4 °C. These were maintained by regular subculturing.

### Biosynthesis of silver nanoparticles

All 20 strains were screened for the biosynthesis of AgNP through the process hereby described. From the stock cultures grown in MEA, a 6 mm diameter disk from the peripheral area of the colony was transferred into a new Petri dish containing malt-glucose-yeast and peptone (MGYP) medium (3.0 g/L malt extract, 10.0 g/L glucose, 3.0 g/L yeast extract, 5.0 g/L peptone and 15 g/L agar) and incubated for 7 days at 30 °C in the dark. Fungal biomass was obtained by inoculating 5 culture disks (6 mm diameter) of each strain in Erlenmeyer flasks containing 100 mL of MGYP broth (3.0 g/L malt extract, 10.0 g/L glucose, 3.0 g/L yeast extract, and 5.0 g/L peptone). Cultures were incubated in an orbital shaker (Quimis, Brazil), for 120 h at 30 °C and 200 rpm. Biomass was then harvested by filtration through Whatman filter paper Grade 3 and was washed three times with sterile distilled water. Wet fungal mycelia (10 g) were suspended in 100 mL of sterilised distilled water and incubated at 30 °C with agitation (200 rpm) for 72 h. After this period, cell-free filtrate was collected by filtration through Whatman filter paper Grade 3. Suspensions were filtrated through a 0.22 µm filter (Millipore) and treated with a silver nitrate (AgNO_3_) solution (1 mM), followed by incubation at 30 °C with agitation (200 rpm), for 120 h in the dark.

### UV–vis absorption spectra (UV–vis)

UV–vis is a widespread method of detection of AgNP (Chan and Don 2012). When bioreduction of AgNP occurred, a change in colour was observed in the AgNO_3_ solution, which turned from yellow into brown. This effect has been reported as an indicator of surface plasmon resonance (SPR) of AgNP (Chan and Don 2013). The position of the plasmonic band detected on the solutions of metallic nanoparticles is dependent on several parameters such as: size, shape, and polydispersity of particles. And, the more the narrow is the band, the bigger is the uniformity index of distribution according to AgNPs size (Becaro et al. 2015). Even though, there was no monitoring of the increase in absorbance until its maximum value, it has been described in the literature that the incubation period used in this study allows for the detection of maximum absorbance, implying the maximum concentration of synthesised AgNPs (Muthukrishnan et al. 2015). The UV–visible spectra of this solution was then recorded on UV–Vis Hitachi U-2000 spectrophotometer (Hitachi, Japan) in a range between 200 and 800 nm.

### Transmission electron microscopy (TEM) and energy-dispersive X-ray analysis (EDX)

The size and shape of the synthesized AgNP were also determined by transmission electron microscopy (TEM) as described by Singhal et al. (2011). A JEOL electronic microscope (model JEM-2100) operated at 200 kV was used for TEM analysis. The average nanoparticle sizes were measured by counting approximately 100 nanoparticles in different regions of each sample, which were then used for the construction of histograms and determination of the average size of the nanoparticles. Energy dispersive X-ray spectroscopy (EDX), model JEOL-JSM 5410 LV (JEOL, USA). To prepare each sample, AgNP were sonicated for 5 min, and a drop of a diluted sample was placed onto a carbon-coated copper grid for analysis.

### X-ray diffraction analysis (XRD)

X-ray diffraction was carried out using a Rigaku, Miniflex II diffractometer (Rigaku, Brazil), equipped with Cu Kα (0.15406 nm) at 40 kV and 30 mA. The diffractograms were recorded over the range 20–90 angles. Lyophilized nanoparticles were placed on a glass grid containing silicon substrate for XRD analysis.

### Size and distribution analysis

For the aqueous suspension containing the AgNP, previously filtered through a 0.22 μm filter, the size distribution and average size of the synthesized AgNP were determined by Dynamic Light Scattering (DLS), Zetasizer Nano ZS90 (Malvern Instruments, UK).

### Nitrate reductase activity

Nitrate reductase activity in the fungal filtrate was assayed by determining the presence of the extracellular enzyme according to the procedure described by Hamedi et al. (2013). Succinctly, the cell filtrate (5 mL) was mixed with an assay medium (30 mM KNO_3_ and 5% propanol in 0.1 M phosphate buffer pH 7.5) in a 1:1 (v/v) proportion and incubated at 30 °C, in the absence of light for 1 h. A sulphanilamide (SA) solution and a *N*-(1-naphthyl) ethylene diamine dihydrochloride (NEED) solution were added to the mixture. The released nitrites on the assay medium then reacted with the SA and NEED solutions and converted into a pink azodye. The absorbance of the resultant pink solutions was measured by UV–visible spectrophotometry, at 540 nm. The enzyme activity of the fungal cell-free filtrate was determined based on the increase in nitrite content of the solution over 1 h and expressed as nmol nitrite/h mL.

### Antibacterial assay of silver nanoparticles

The antimicrobial activities against the Gram-positive bacteria: *Staphylococcus aureus* IPT246; and the Gram-negative bacteria: *Escherichia coli* IPT245, and *Pseudomonas aeruginosa* IPT322, were determined by agar plate well diffusion assay. Bacteria were cultured in Mueller–Hinton agar (MHA; 2.0 g/L beef extract, 17.5 g/L casein hydrolysate, 1.5 g/L starch, and 17 g/L agar), MHA, applying 100 μL of an initial inocullum (10^6^ CFU/mL) of each strain in the agarised media and uniformly spreading. Subsequently, 100 μL of a AgNP solution at 1.0, 5.0, 10, 50, and 100 μg/mL concentrations were added into 3 mm diameter wells, cut out in the centre of the plate, and incubated at 37 °C for 24 h. Streptomycin solutions (100 μL) were used, in the same concentration values, as positive control, and water as negative control. After incubation, the zones of inhibition were measured. The assays were performed in triplicate.

## Results

### Silver nanoparticles biosynthesis

The synthesis of AgNP was detected by UV–vis, and from all the strains screened, only four had the aptitude to synthesize AgNP. Those were identified at IPT as: *Rhizopus arrhizus* IPT1011, *Rhizopus arrhizus* IPT1013, *Trichoderma gamsii* IPT853, and *Aspergillus niger* IPT856. The development of a brown colour was indicative of the formation of AgNP by ion reduction. AgNP were detected by the absorbance peak at 418–430 nm after 72 h of incubation.

### UV–vis absorption spectra (UV–vis)

**Fig. 1:**
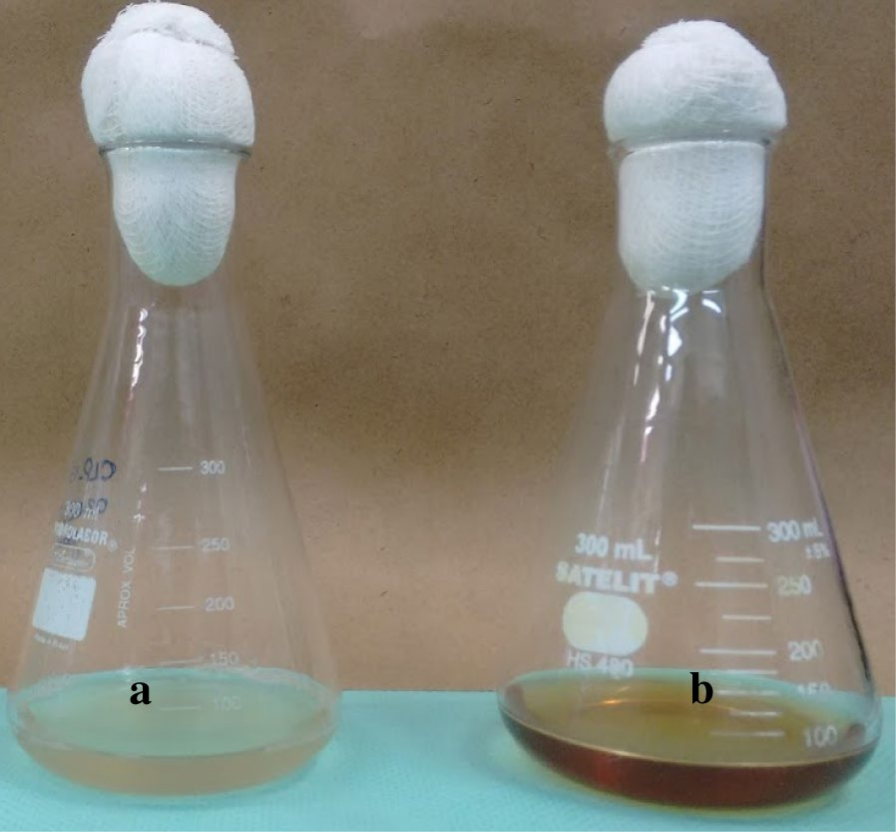
Colour change, for the strain IPT 1013, between **a** initial and **b** finishing step of the mycogenic reaction

The intense dark brown colour of the fungal filtrate occurred after the addition of AgNO_3_, after a 24 h time period as seen on the representative image (Fig. 1). After 72 h, the maximum absorption values of the analysed strains were: 418 nm (IPT1011), 420 nm (IPT1013), 426 nm (IPT853) and 430 nm (IPT856). For all the filtrates with no addition of AgNO_3_, a peak between 279 and 285 nm was detected, which has been previously described as being common for biomolecules (Gopinath and Velusamy 2013).

### Transmission electron microscopy (TEM) and energy-dispersive X-ray analysis (EDX)

**Fig. 2:**
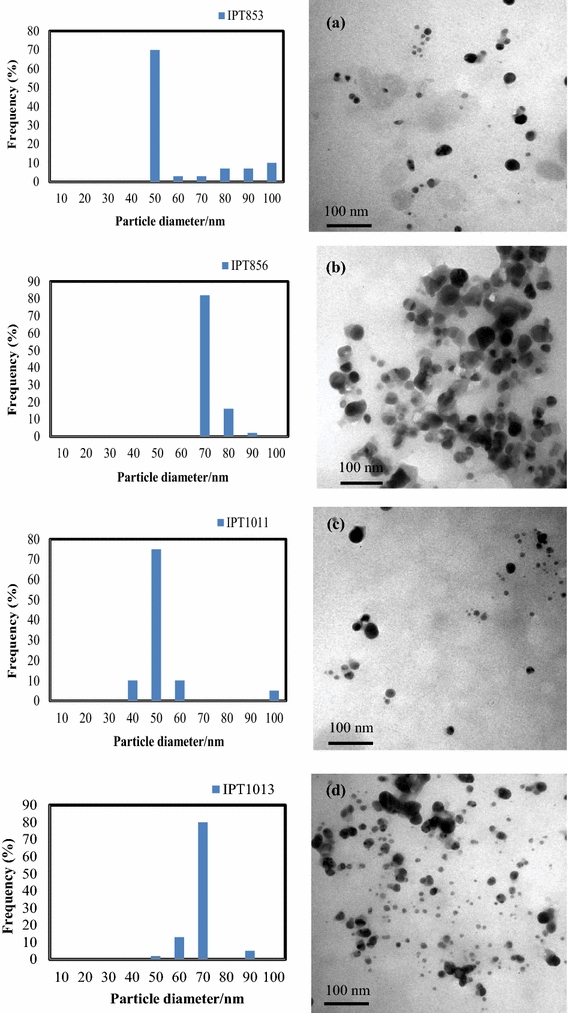
TEM images and respective histograms of AgNP synthesized by: **a** IPT853, **b** IPT856, **c** IPT1011, and **d** IPT1013

All the biosynthesised AgNP presented a spherical shape and their respective histograms are shown in Fig. 2. The data observed in the histograms, is presented in Table 1.

**Table 1:** Size and charge characteristics of synthesized AgNP

Nanoparticles	SEM average size (nm) (images not shown)	TEM		DLS average size	Zeta potential (in water) (mV)
		Average size (nm)	AgNP with the average size (%)		
IPT853	55.1	50	70	63.8	−16.53
IPT856	66.7	70	80	79.3	−28.40
IPT1011	31.6	50	70	53.0	−19.24
IPT1013	36.4	70	80	82.6	−21.35

**Fig. 3:**
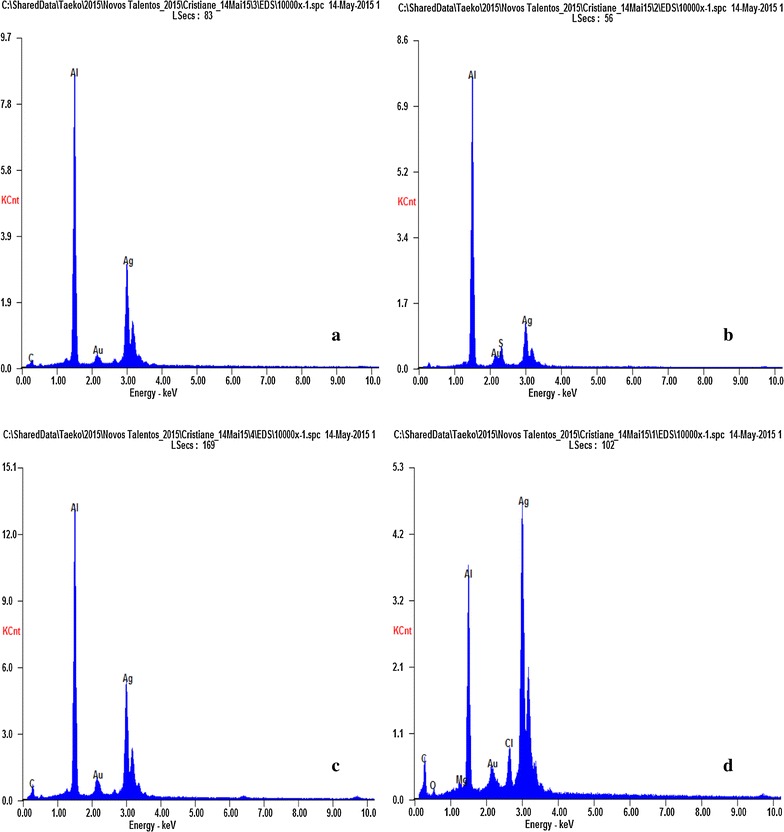
EDX spectra of AgNP synthesized by: **a** IPT853, **b** IPT856, **c** IPT1011, and **d** IPT1013

EDX characterisation has shown absorption of strong silver signal (Fig. 3). The absorption peak at 3 keV is typical of crystalline nature of the AgNP.

### X-ray diffraction analysis (XDR)

**Fig. 4:**
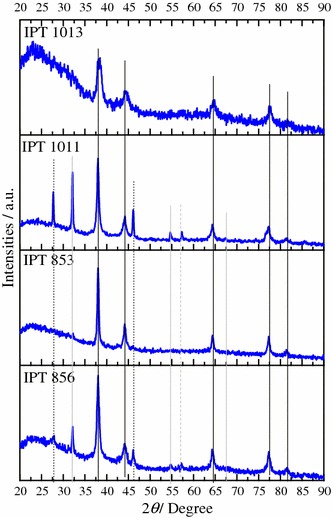
XRD patterns of synthesized AgNP. *Black line* is Ag, *grey line* is AgO, *grey dash* is Ag_2_O_2_ and *black dotted line* is C

Regarding XRD analysis (Fig. 4), it was possible to observe a well-defined face-centered cubic (FCC) structure of Ag for all materials, at 38°, 44°, 64.5°, 77°, and 82°, corresponding to planes (1 1 1), (2 0 0), (2 2 0), (3 1 1), (2 2 2) respectively and lattice parameter at 0.409 nm according to JCPDF # 04-783.

Using the Debye–Scherrer method (Cullity, 1967), the measured average crystallite size of AgNP for each strain was: 11 nm for IPT853, 12 nm for IPT856, 16 nm for IPT1011, and 8 nm for IPT1013.

The AgNP synthesized by the strain IPT1013, presented the most deformed pattern by low structured carbon support (large peak ≈25°) (Wang et al. 2013; Modibedi et al. 2011) due to smaller crystallite size. For IPT853 and IPT1011 AgNP, it was possible to observe two carbon phases structured at 26° and 46° (JCPDF # 18-311), silver oxides (AgO; JCPDF # 76-1489) at 32° and 55°, and Ag_2_O_2_ (JCPDF # 51-945) at 57°. These oxides presented a crystallite size around 4 nm.

### Size and distribution analysis

The sizes, distribution and polydispersity index (PDI) for AgNP (Table 1) were determined for the strains capable of biosynthesis.

The results obtained through TEM were similar to the ones obtained by DLS. Similar to what was described by Singhal et al. (2011), the size of the metal nanoparticles determined by DLS was slightly larger when compared to the particle size measured from TEM micrographs. According to the authors, this happens because DLS measures the hydrodynamic radius.

### Nitrate reductase activity

**Fig. 5:**
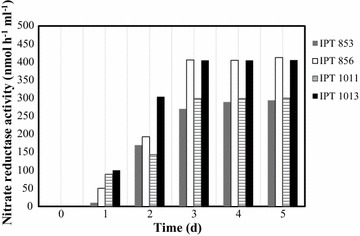
Nitrate reductase activity detected on the fungal cell-free filtrate from the strains capable of AgNP synthesis

Nitrate reductase activity of the culture supernatants for the strains IPT853, IPT856, IPT1011, and IPT1013 was detected by the nitrate reductase assay, and analysed over a period of 5 days (Fig. 5). No activity was detected for the remaining strains tested in this study.

Nitrate reductase activity of the isolates capable of nanoparticle synthesis supports the hypothesis of enzymatic reduction of silver nitrate into silver nanoparticles (Hamedi et al. 2013; Saifuddin et al. 2009).

### Antibacterial activity of AgNP

**Fig. 6:**
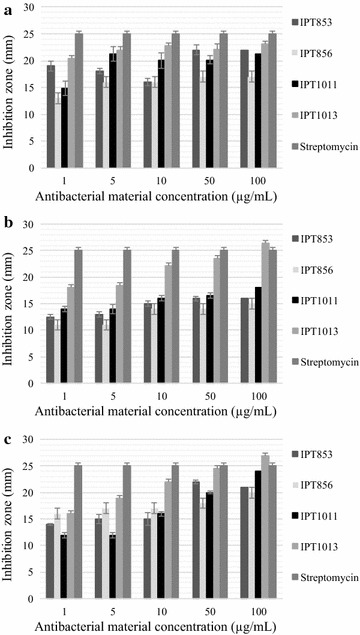
Growth inhibition of: **a** *E. coli*, **b** *S. aureus*, and **c** *P. aeruginosa* by different concentrations AgNP of the four synthesizing strains and by streptomycin (used as positive control)

Antibacterial activity of biosynthesized AgNP was evaluated by growth inhibition in agar plates. The AgNP showed inhibition of growth of *E. coli* (Fig. 6a), *S. aureus* (Fig. 6b) and *P. aeruginosa* (Fig. 6c).

Furthermore, and as expected, no inhibition was detected when using the negative control—water (data not shown).

## Discussion

Bioprospection on different and less commonly studied environments allows us to analyse the microbial diversity and encounter microbes specialised in certain bioproducts, like metal nanoparticles. When compared with physical–chemical methods, the microbial biosynthesis of nanoparticles by microorganisms is faster, cheaper, more effective, and without the involvement of hazardous chemicals (Durán et al. 2016; Rahimi et al. 2016). In this study, an initial batch of 20 fungal strain, isolated from sugar cane plantation soil, was screened for its biogenic capacity of producing AgNP by reducing silver nitrate, and four fungal strains (*Rhizopus arrhizus* IPT1011, *Rhizopus arrhizus* IPT1013, *Trichoderma gamsii* IPT853, and *Aspergillus niger* IPT856) were detected to be capable of biosynthesizing AgNP. According to our measurements, the four selected strains were capable of extracellular biosynthesis of AgNP of uniform size and round-shaped, with diameters in the range of 30–100 nm. Extracellular secretion of enzymes by fungi allows to easily recover those enzymes, which in our study were then used for nanoparticles synthesis, turning this into an effortless biological method.

The exact mechanism of AgNP synthesis by fungi is not yet clearly known but previous studies have indicated that NADH and NADH-dependent nitrate reductases are important factors in the biosynthesis of metal nanoparticles (Ahmad et al. 2003; Hamedi et al. 2013). In the present study, the activity of nitrate reductase was measure for all 20 strains, capable and non-capable of biosynthesizing AgNP. The lack of activity in the cell filtrates for the strains that did not show reduction of silver nitrate supports the hypothesis of enzyme based biosynthesis.

Furthermore, the differences in intensity, shown in Fig. 4, are linked to the structuring process of the silver crystal. The more regular and larger the crystallite formed, the greater the intensity and the smaller the width of the Brag peaks. This structure of the crystals is dependent on the chemical environment, pressure, temperature and time; as these last three factors were constant for all the samples, we can then say that the chemical environment of the fungi was responsible for the differentiation of the structures obtained, which is linked to the Nitrate reductase activity profiles. This activity (Fig. 5), is very low in the initial moments for the strain IPT853 (*Trichoderma gamsii*), while for IPT1011 (*Rhizopus arrhizus*) it is more pronounced. We know that the initial moments of nucleation of the nanoparticles dictate the structure and stability of the crystal, and these differences are what caused the appearance of the oxides in other samples. However, all profiles detected by XRD reflect Ag, AgO, AgO_2_ and carbon particles, with no other contaminant species crystallized.

The AgNP produced by the *Rhizopus arrhizus, Trichoderma gamsii* and *Aspergillus niger* strains in this study were found to be active against *E. coli*, *S. aureus, and P. aeruginosa*. Multiple bactericidal mechanisms can act in synergy to confer a broad spectrum of activity against different types of bacteria. It is known that the antimicrobial activity of AgNP is due to the formation of insoluble compounds by inactivation of sulfhydryl groups in the cell wall and disruption of membrane bound enzymes and lipids resulting in cell lysis (Dorau et al. 2004). And it has also been reported that the process may involve the binding of AgNP to external proteins to create pores, interfering with DNA replication or forming reactive oxygen species (ROS) such as hydrogen peroxide, superoxide anions, and hydroxyl radicals (Duncan 2011; Durán et al. 2016; Jung et al. 2008).

The AgNP of smaller dimensions in this study, produced by *R. arrhizus,* were shown to be the most efficient against the bacteria tested. In fact, several studies have shown that AgNP activity is strongly dependent on the NP size (Wu et al. 2014; Tamayo et al. 2014; Rahimi et al. 2016).

The correlation between the bactericidal effect and AgNP concentrations is bacterial class dependent (Chernousova and Epple 2013). Just like in previous studies (Zhang et al. 2014), *E. coli* was more affected by AgNP than *P. aeruginosa* and the inhibitory effect on the growth of *S. aureus* was less marked than in *E. coli* as previously found by Wu et al. (2014). This strengthens several previous investigations (Pal et al. 2007; Fayaz et al. 2010; Devi and Joshi 2014), that found that Gram-positive and -negative bacteria have different susceptibility to AgNP, probably due to differences in their membranes and cell walls (Feng et al. 2000).

The fact that bacterial resistance to elemental silver is extremely rare (Silver 2003) emphasizes the increased interest in using AgNP as potent antimicrobial agent in biomedical applications. The increase in publications on this topic, like our own research, will benefit future research and development of cost-effective metal nanoparticles production, with desirable therapeutic effects.

The presence of nitrate reductase in the supernatant supports the hypothesis of its strong influence on the mycogenic synthesis process. All the four biosynthesised AgNP were characterised and showed potential antimicrobial activity noted through growth inhibition of several bacterial species. Furthermore, we observed a direct relation between the concentration of AgNP and antimicrobial capacity.

Further analyses are needed to fully understand the mycogenic synthesis process and the mechanisms involved in AgNP production by these strains. Nevertheless, our study proves the importance of exploring more environments and analyse their microbial community to discover novel and/or better bio-products. This will have high impact on society and health, as the world is facing a massive increase of microbial resistance to most of the known and commercially available drugs and antibiotics.

## Abbreviations

IPT: Instituto de Pesquisa Tecnológica do Estado de São Paulo
AgNP: silver nanoparticles
SPR: surface plasmon resonance
TEM: transmission electron microscopy
EDX: energy-dispersive X-ray analysis
XRD: X-ray diffraction analysis
